# Recognizing Psychiatric Comorbidity With Reading Disorders

**DOI:** 10.3389/fpsyt.2018.00101

**Published:** 2018-03-27

**Authors:** Robert L. Hendren, Stephanie L. Haft, Jessica M. Black, Nancy Cushen White, Fumiko Hoeft

**Affiliations:** ^1^Division of Child and Adolescent Psychiatry, Department of Psychiatry, Weill Institute for Neurosciences, University of California San Francisco, San Francisco, CA, United States; ^2^Dyslexia Center, University of California San Francisco, San Francisco, CA, United States; ^3^School of Social Work, Boston College, Chestnut Hill, MA, United States; ^4^Department of Pediatrics, University of California San Francisco, San Francisco, CA, United States; ^5^University of California Multi-Campus Precision Learning Center (PrecL), San Francisco, CA, United States; ^6^Haskins Laboratories, New Haven, CT, United States; ^7^Department of Neuropsychiatry, Keio University School of Medicine, Tokyo, Japan

**Keywords:** specific learning disorder, developmental dyslexia, comorbidity, mental health, neurodevelopmental

## Abstract

Reading disorder (RD), a specific learning disorder (SLD) of reading that includes impairment in word reading, reading fluency, and/or reading comprehension, is common in the general population but often is not comprehensively understood or assessed in mental health settings. In education settings, comorbid mental and associated disorders may be inadequately integrated into intervention plans. Assessment and intervention for RD may be delayed or absent in children with frequently co-occurring mental disorders not fully responding to treatment in both school and mental health settings. To address this oversight, this review summarizes current knowledge regarding RDs and common comorbid or co-occurring disorders that are important for mental health and school settings. We chose to highlight RD because it is the most common SLD, and connections to other often comorbid disorders have been more thoroughly described in the literature. Much of the literature we describe is on decoding-based RD (or developmental dyslexia) as it is the most common form of RD. In addition to risk for academic struggle and social, emotional, and behavioral problems, those with RD often show early evidence of combined or intertwined *Diagnostic and Statistical Manual of Mental Disorders, Fifth Edition* childhood disorders. These include attention deficit hyperactivity disorder, anxiety and depression, disruptive, impulse-control, and conduct disorders, autism spectrum disorders, and other SLDs. The present review highlights issues and areas of controversy within these comorbidities, as well as directions for future research. An interdisciplinary, integrated approach between mental health professionals and educators can lead to comprehensive and targeted treatments encompassing both academic and mental health interventions. Such targeted treatments may contribute to improved educational and health-related outcomes in vulnerable youth. While there is a growing research literature on this association, more studies are needed of when to intervene and of the early and long-term benefits of comprehensive intervention.

## Introduction

Despite a strong reciprocal association between reading disorder (RD) and mental disorders in young people (1), their co-occurrence is often under-recognized and under-treated resulting in less than optimal outcomes in all areas including emotional outcomes. Difficulties with comorbidities may continue into adulthood (2). Recognition of RD by health-care professionals is important—the prevalence of dyslexia (decoding-based RD; the term RD is used from hereon) is approximately 5–10% of all children depending on the study across languages, cultures, and writing systems (3).

**Figure 1:**
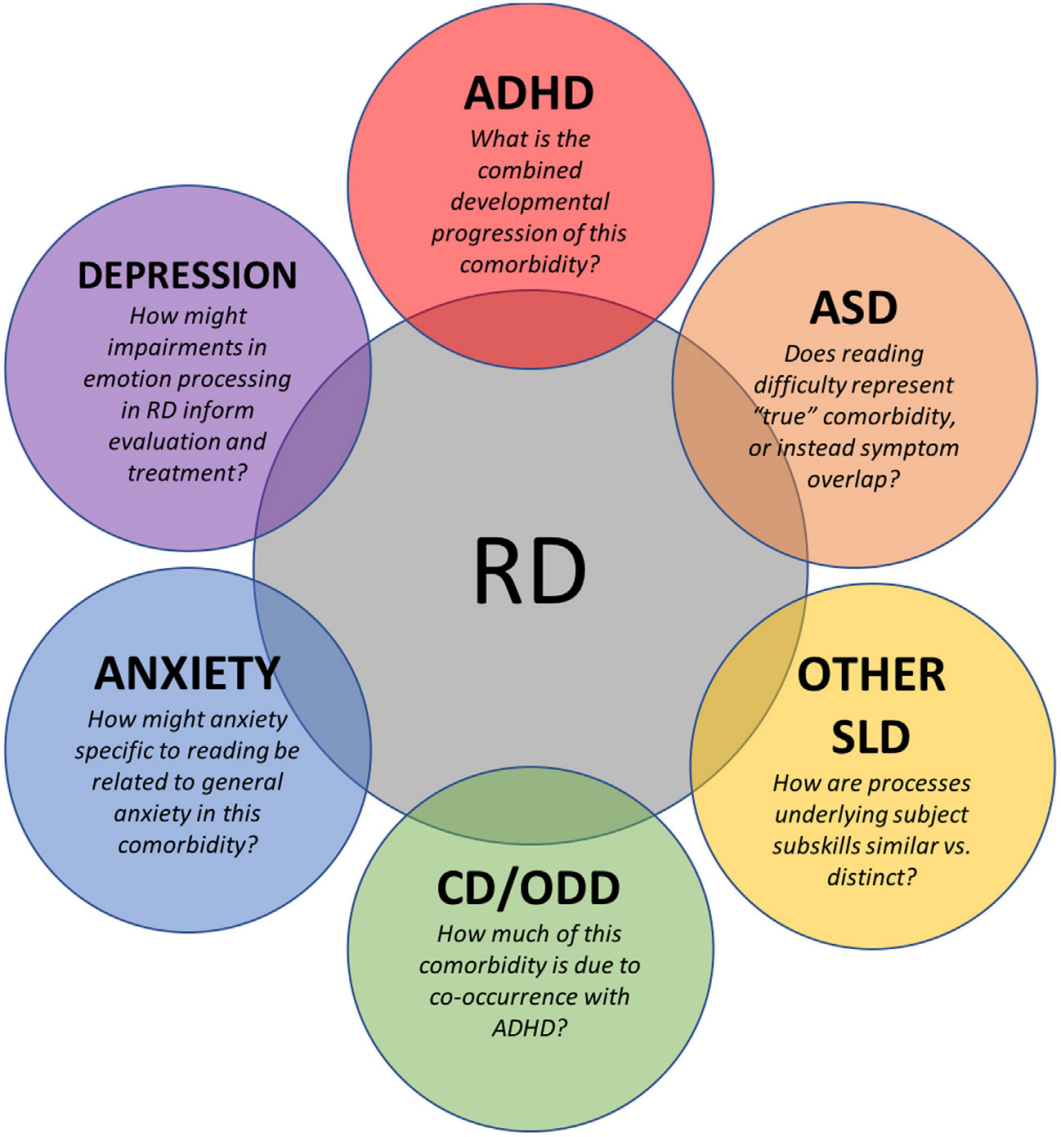
Current issues, areas of investigation, and suggestions for future research in conditions commonly occurring with RD in children. RD, reading disorder; ADHD, attention deficit hyperactivity disorder; ASD, autism spectrum disorder; SLD, specific learning disorder; CD, conduct disorder; ODD, oppositional defiant disorder.

In an effort to bridge the recognition gap between RD and associated mental disorders, we review RD along with other co-occurring *Diagnostic and Statistical Manual of Mental Disorders, Fifth Edition* (DSM-5) mental disorders. We also review literature that describes best practice interventions for children with RD and comorbid disorders and identify areas where stronger research is important (Figure 1). Our overarching goal is to increase the awareness of health professionals to disorders of reading that overlap or are confused with mental conditions and disorders.

DSM-5 defines RD, within neurodevelopmental disorders, as a type of specific learning disorder (SLD) with impairment in reading that is characterized by problems with word reading accuracy, reading fluency, and reading comprehension that are not the result of sensory impairments, neurological disorders, intellectual disabilities, or inadequate educational instruction (4). The impairments in word reading accuracy or reading fluency are also noted as developmental dyslexia in the literature. RD is often associated with difficulties in phonological awareness (awareness of sounds of a language, i.e., phonemes, to process spoken and written language), lexical fluency (i.e., rapid naming of common items), letter (sound) knowledge, grapheme–phoneme association, which are predictive of later decoding and word reading. Oral language, vocabulary, and executive function on the other hand are generally more predictive of reading comprehension. Difficulties in reading comprehension may be a result of reduced reading experience that can impede growth of vocabulary and background knowledge in those with decoding-based RD (5). However, it is possible for children with specific reading comprehension deficits to have intact decoding skills (6).

## Methods

We used two electronic databases (PubMed and PsycInfo) in order to review prevalence and characteristics of RD’s comorbidity with other psychiatric disorders. Searching for relevant articles from the past 20 years (1997–2017), we used the keywords such as “dyslexia,” “reading disability,” “reading disorder,” “learning disability,” “learning disorder” along with “comorbid,” and/or terms pertaining to other specific DSM-5 disorders [e.g., “autism and Autism Spectrum Disorder (ASD),” “Attention Deficit Hyperactivity Disorder (ADHD),” “anxiety,” “depression,” “conduct disorder,” etc.]. We primarily selected articles with a focus on child populations (individuals under the age of 18 years) and a specified diagnosis of RD.

### Overview of RD

#### Etiology

Reading disorder results from a constellation of genetic and environmental risk factors and their interactions and not a single underlying cause. The estimated heritability rate of RD is approximately 50–70% (7, 8). Several susceptibility genes have been identified (8), though each explains only a small fraction of variance, suggesting the involvement of other mechanisms including polygenicity, epistasis, and epigenetics, in RD (9). Neuroanatomical anomalies in both gray and white matters shown to be causally related to RD (10) are observed in areas and networks associated with phonological, orthographic, and articulatory processing (11–13). Additionally, work in neural oscillations as well as neurochemistry shows deficits related to sensory processing, particularly auditory discrimination, in individuals with or at-risk for RD (14, 15). Within the context of comorbidity, the pathophysiology often overlaps between RD and co-occurring mental disorders. For example, RD shares common risk genes with ADHD (16, 17). In ASD, links to language impairment (LI) such as specific language impairment (SLI) have been made (18), which in turn may be associated with RD risk genes (19). Neuroimaging studies of RD comorbidity with mental disorders are currently limited but hold promise for elucidating shared versus differential etiologies. For instance, one neuroimaging study found distinct neural biomarkers for children with dyslexia, ADHD, and age-matched controls in auditory neuroanatomy, physiology, and behavior (20).

#### Early Characteristics

In those at-risk for developing decoding-based RD, deficits in pre-literacy skills (e.g., phonological awareness, letter identification and letter-sound knowledge, and rapid naming) are observed (21). A growing body of research also implicates non-linguistic, domain-general abilities in early literacy acquisition and RD, such as visual attention (22) and executive functions (23). Decoding-based RD is often noticed initially in kindergarten or first grade when children are first exposed to formal reading instruction and may be diagnosed between 2nd and 4th grade, depending on the educational system, parents, caregivers, and teachers. RD of reading comprehension tends to be identified later as the demands of reading increases from learning to read to reading to learn, unless children are initially diagnosed in earlier years with SLI. Though profiles of specific comorbidities will be discussed in later sections, the general pattern is that RD in combination with a comorbid condition results in greater impairment.

#### Assessment and Diagnosis

In order to obtain a formal diagnosis of RD, a child must undergo a battery of tests that are administered by a qualified professional (diagnostician qualifications vary by state). Careful consideration of the potential for co-occurring disorders or impairments and other interacting factors is critical for ensuring accurate diagnoses to inform recommendations for intervention or treatment—and for predicting prognosis. For example, it would be important to determine whether a child presents with ADHD and has a secondary difficulty in reading or presents with RD that results in inattention.

Prior to assessment, it is important to obtain the child’s family, developmental, and educational history. Sensory issues (e.g., impairment in vision or hearing), home and school literacy environment, native language (e.g., English learners who lack English proficiency) that may affect reading should be ruled out as causes of difficulty; at the same time, it must be kept in mind that the presence of these issues do not necessarily preclude existence of RD. Parental self-report scales of reading and attention difficulties may be useful for identification of adults at-risk for these difficulties, since at-risk parents may confer risks on their children for related problems (24).

## Comorbid Mental Disorders

### Neurodevelopmental Disorders

#### Attention Deficit Hyperactivity Disorder

Attention deficit hyperactivity disorder and RD are recognized as frequently comorbid disorders (Table 1). ADHD involves an unremitting pattern of inattention and/or hyperactivity–impulsivity that results in functional impairment (4). RD often involves attention difficulties, sometimes representing overlooked and undertreated ADHD (25), which can contribute to academic underachievement (26). Subgroups of children with RD show attention-related impairments (e.g., deficits in alertness, covert shift of attention, divided attention, flexibility, and visual search) (27). In experimental work, researchers have shown attention-related deficits in RD in both the auditory (attention shifting) (28) and visual domains (which some argue is an independent contributor to phonological deficits) (29).

**Table 1:** An overview of comorbid conditions that commonly occur with reading disorder (RD) as well as their shared symptoms and risk mechanisms.

Comorbid condition	Features of comorbid group	Shared risk with RD
Attention deficit hyperactivity disorder (ADHD)	Inattention (30, 31) in auditory (28) and visual (29) Deficits in processing speed, verbal working memory, phonological short-term memory, naming speed, and central executive processes (32, 33)	Shared risk genes (*KIAA0319* and *DCDC2*) (17) Shared structural and functional neural abnormalities (33) Environmental factors (smoke and miscarriage) (16)
Autism spectrum disorder	Impaired reading comprehension (34)	Shared risk genes (*MRPL19*) (19) Comorbidity with language impairment (35)
Disruptive, impulse-control, and conduct disorders	Externalizing behavior (36, 37)	Shared cognitive risk in working memory deficit (38) Comorbidity with ADHD (39–41) Deficits in verbal processing/language skills (38, 42)
Anxiety and depressive disorders	Poor self-esteem (43) Internalizing psychopathology (44)	Negative academic/social experiences (45) Shared familial risk factors (46)
Other specific learning disorders	Internalizing psychopathology (47) Handwriting deficits (48)	Shared cognitive risk in working memory, semantic memory, and verbal processing deficits (49) Deficits in rhythmic organization (48)

Approximately 20–40% of children with the inattentive subtype of ADHD have RD (50, 51) and 20–40% of those with RD have ADHD (33). The relationship between ADHD symptoms and reading is found to be predominantly in the inattentive type (30, 31). Neuropsychological profiles of RD and ADHD comorbid groups include deficits in processing speed, verbal working memory, phonological short-term memory, naming speed, and central executive processes (32, 33). A strong explanation for this comorbidity proposes that multiple predictors of each disorder have shared or overlapping genetic (16, 17), as well as neural and cognitive risk factors. A proposed subgroup of ADHD, “sluggish cognitive tempo,” characterized by difficulty sustaining attention, daydreaming, lethargy, and physical underactivity, is thought by some to represent a possible link to RD (52) using electrophysiological (53) and pharmacological (54) evidence. Imaging studies, however, have generally found distinct patterns of structural and functional abnormalities among RD and ADHD, most often examined separately (55).

The research on comorbidity between RD and ADHD is extensive—for reviews, see Ref. (17, 33, 56). However, the combined developmental progression of RD + ADHD is not well studied. Further research is needed of the overlap or intertwined nature of the two disorders and the influence of this potential co-contribution during the development of reading.

#### Autism Spectrum Disorder

There are limited studies of ASD + RD children, and the prevalence of RD reported in ASD children ranges considerably from 6 (57) to 30% (58). One consideration in discussing co-occurring ASD and RD is differentiating between “true” comorbidity and symptom overlap—a recurring issue in child psychopathology. In both ASD and RD, there are documented impairments in reading comprehension, language, and visual/auditory processing. However, simply counting these overlaps in cognitive symptoms in ASD and RD may lead to false recognition of comorbidity. On the other hand, failure to recognize a comorbid RD could result in inadequate treatment with negative academic consequences.

Reading disorder and ASD might not be considered “true” co-occurring disorders because the reading difficulty in ASD is not a decoding or phonics problem. Numerous studies report reading comprehension deficits in children with ASD that are discrepantly low with their intelligence [see Ref. (59) for meta-analysis], which typically do not fall under decoding-based RD. In fact, though reading comprehension impairment in ASD children is well-documented (34), ASD children often show intact and sometimes even precocious abilities in word reading, non-word decoding, and text reading accuracy (60). These findings suggest that the reading deficits observed in ASD are different than that of a child with decoding-based RD, which are characterized by phonological deficits that may lead to impaired reading comprehension.

One way to further explore if ADHD + RD represent a “true” comorbidity is to examine the possibility of shared etiological factors. One explanation for shared reading problems is co-occurring LI. LI is prevalent in both ASD (61) and RD (62), and studies suggest that the presence of reading comprehension deficits in children with ASD is mediated by language ability (35, 61). These behavioral results are supported by a study showing that genes contributing to general language skills are shared among dyslexia, ASD, and LI (19). However, LI is certainly not the sole contributor to reading problems in ASD—some literature shows a correlation between social abilities and reading comprehension in ASD (60). In other words, the behavioral manifestation of reading impairment in ASD and RD originates at least partly from different mechanisms; however, longitudinal and family studies are needed to further explore how the disorders may be related. As discussed in the treatment section, the underlying explanation for the RD and ASD comorbidity has important implications for how comorbid RD is treated in children with ASD, and further study is needed to determine the most effective differential interventions.

#### Other SLDs

Historically, the subtypes of SLDs have been viewed from an academic-subject approach—for example, the DSM-4 had distinct categories for RD, mathematics disorder (MD or dyscalculia), disorder of written expression (dysgraphia), and learning disorder not otherwise specified. The DSM-5 moved away from these categories in including RD, MD, and dysgraphia together under the label of “Specific Learning Disorder” with specifiers for the area of impairment. These areas of impairment can be further broken down into component subskills in the areas of reading (e.g., word reading accuracy, reading fluency, and reading comprehension), mathematics (e.g., number sense, calculation, and math reasoning), and writing (e.g., spelling, grammar, and written expression). In a move from a categorical to a dimensional approach, research has sought to identify comorbidities among the SLDs through the lens of shared versus unique cognitive processes that might underlie them.

Reading disorder and MD have a comorbidity of approximately 40% (63), and this co-occurrence is associated with greater impairment on measures of internalizing psychopathology and academic functioning (47). Although RD and MD are generally accepted to have unique neurocognitive profiles, researchers have pointed to shared cognitive processes in working memory, semantic memory, and verbal processes that may explain the high comorbidity (49). One recent paper applied a cluster analysis to children with SLD to identify associations between cognitive clusters and SLD subtypes. Results showed that impaired subskills of each domain were associated with different clusters—for example, math and text reading speed were most strongly associated with a cluster involving cognitive processing, while text comprehension was more linked to the verbal abilities cluster (64). This approach is promising in recognizing the heterogeneity within RD and MD themselves, as well as adopting a dimensional approach to highlight shared cognitive deficits.

Reading disorder has also been shown to co-occur with dysgraphia. Comorbidity rates between RD and dysgraphia are difficult to determine, yet the correlation of word reading and writing performance is shown to be around 70% (65). Although RD and dysgraphia are shown to have differences in brain bases for written language tasks (66), they exhibit shared behavioral deficits in rhythm, which is required for both reading and writing (48). Most researchers have explained the overlap of dysgraphia and dyslexia by highlighting learning to read and learning to spell as “two sides of the same coin” (65). Phonological awareness, visual attention, working memory, and auditory processing play predictive roles in both reading and writing (67).

Further research is warranted on examining comorbidities between RD and other SLDs from a process perspective. In particular, studies should examine trajectories of impairment in these cognitive processes from before the onset of formal schooling to adult years when “compensation” for deficits may have occurred.

### Disruptive, Impulse-Control, and Conduct Disorders (CDs)

Children with RD can exhibit comorbidities in the disruptive, impulse-control, and CD categories of the DSM-5 including CD and oppositional defiant disorder (ODD) (4). Most of the existing literature focuses on associations between RD and behavioral problems or disorders in general, though specific links between RD and diagnosed CD (68) have been established. It is not clear, however, how much of the higher incidences of externalizing behavior among children with RD precedes RD or is the emotional result of it (36, 37). Although more recent studies have found that reading difficulties often precede behavioral problems, results do not necessarily support a direct causal pattern between the two conditions. Instead, conduct and behavioral issues in RD children are exhibited across both academic and non-academic settings and appear more independent of reading problems (69, 70). These findings are important in implying that interventions for RD may not treat co-occurring behavioral problems—however, such treatment studies have yet to be conducted and represent an area of needed research.

One promising explanation for the co-occurrence of RD and behavioral disorders is each conditions’ comorbidity with ADHD—ADHD commonly occurs with RD, CD, and ODD (71), and ADHD and RD are associated with higher delinquency severity scores than for either one alone (72). Further support for this explanation comes from studies showing that hyperactivity mediates between reading problems and disruptive behaviors in adolescent populations (39, 73). However, one study of adult forensic patients with RD found a higher level of cognitive impulsivity than those without RD, regardless of ADHD diagnosis (74). An additional explanation for the comorbidity of these conditions with RD involves shared neurocognitive risk factors—for example, children diagnosed with disruptive and CDs are shown to have abnormal language processing (42) and working memory deficits (38), characteristics also shared by children with RD. Taken together, these results suggest that the comorbidity of disruptive, impulse-control, and CDs and RD are at least partially due to each disorders’ co-occurrence with ADHD. Further study is needed to determine how the co-occurrence of RD and CD/ODD may differ with or without the presence of comorbid ADHD. This will inform the most effective timing and nature of interventions to improve outcomes for RD and intertwined behavioral disorders.

### Anxiety Disorders

Children with RD report greater generalized anxiety than their non-RD peers (44), and a meta-analysis has confirmed that LD children and adolescents, including those with RD, have significantly higher scores on anxiety measures than non-LD students (75). This higher rate of anxiety in RD children persists even after controlling for ADHD symptoms (76). In explaining this comorbidity, researchers have proposed a model whereby anxiety distracts from learning and interferes with cognitive processes necessary for reading, leading to potential RD (77). However, researchers have also proposed that reading problems associated with RD can lead to anxiety as a result of the experience of school failure (78). More neurodevelopmental longitudinal studies are needed to investigate these processes, although current evidence suggests that both models have merit, with a bi-directional relationship between anxiety and reading (79).

In order to investigate a potential genetic etiology for the RD-anxiety comorbidity, researchers have studied siblings and twin pairs. One study of monozygotic and dizygotic adult twins found a strong link between anxiety and RD but with no shared genetic cause (45). A separate study demonstrated that siblings of children and adolescents with RD were more than twice as likely to meet criteria for generalized anxiety disorder (GAD), suggesting shared familial risk factors between the two disorders (46). The study also showed marginally significant differences between monozygotic and dizygotic twin pairs in RD cross-concordance with GAD, indicating a small role for genetic risk in the comorbidity between RD and anxiety. Although more work is needed on neural correlates of comorbidity, these genetic studies support the model of a combination of genetic and environmental risk factors in explaining co-occurrence of RD and anxiety.

One area of future research involves distinguishing between comorbidity of RD and general anxiety versus anxiety specific to reading (*reading anxiety*). Reading anxiety as a concept has not been investigated in the literature, but over three decades of research on math anxiety indicate that its neural and behavioral characteristics are related but distinct from general anxiety [see Ref. (80, 81) for reviews on math anxiety]. There is no doubt that RD is commonly comorbid with general anxiety, but investigating the potential presence of reading anxiety could enable more targeted interventions to address co-occurring emotional problems children with RD. Unfortunately, there are currently no measures to assess reading anxiety, representing an area of need in the field.

### Depressive Disorders

In addition to or potentially as a result of anxiety, children and adolescents with RD exhibit higher rates of depression (44, 82), with evidence for a correlation between more severe RD and greater depressive symptoms in younger children (83). Similar to the research on RD and anxiety association, the existence of depression in RD does not appear to be dependent on comorbidity with ADHD (84). Researchers have identified low self-esteem as a symptom of depression in RD as well as a target for intervention (85, 86)—in one study of adolescents with RD, self-esteem predicted 23% of the variation in depression risk (87). Depression and RD exhibit patterns of familial risk and marginally significant genetic contributions similar to that of RD and anxiety (46), suggesting multiple risk factors. The higher incidence of bullying and peer victimization faced by children and adolescents with RD may be an environmental factor that partially explains comorbidity with depression (88) but further study of neurodevelopmental risk factors will likely provide targets for early interventions.

For example, a growing area of research suggests that emotion processing may be impaired in children with RD (89). This impairment has important implications for assessing for comorbid depression and anxiety in RD (44), since deficits in understanding emotions, depressive and anxious symptoms may go underreported. Thus, self-report measures may not be sufficient to assess for comorbid depression and anxiety in RD youth.

### Other Disorders and Conditions

Reading disorder can sometimes co-occur with other DSM-5 categories, though these appear to be less investigated than the aforementioned conditions. Although RD is not listed as a common comorbid condition in the category of *sleep-wake disorders* and *vice versa*, a recent exploratory study found a significantly greater frequency of sleep disorders in RD children compared to controls (90). Given that a prior neurophysiological study showed an association between sleep activity and reading abilities in RD children (91), evaluation of sleep may be an important factor to consider in RD treatment and management.

Reading disorder may also co-occur with disorders more commonly appearing in adulthood. For instance, one study of *substance-related and addictive disorders* showed that out of a sample of adults with addiction issues, 40% had RD (92). However, a separate study reported significantly lower substance use history in RD versus non-RD university students (93). Future research is therefore needed to draw conclusion about the comorbidity of RD and substance abuse. Similarly, due to the rarity of early onset *schizophrenia*, RD and schizophrenia have not been shown to co-occur in children, but one study found that 70% of adult patients with schizophrenia met criteria for RD (94). However, this finding may be confounded in part by reduced educational and occupational outcomes (94), as well as IQ changes that may occur with progression of schizophrenia (95). Finally, a form of RD can occur in patients with the neurocognitive disorder of *dementia* (96) and may share susceptibility genes (97), though this is only observed in adult populations.

## Treatment

A challenge in treating comorbid conditions is whether to target both conditions simultaneously or to treat one condition to see if benefit in the other condition results. However, there is a gap in the literature of evidence-based strategies for treating RD with comorbid conditions, likely because investigations of treatments often intentionally exclude individuals with comorbidities. This is further complicated by the fragmented approach to treatment a child with RD may receive. For example, an educator may focus on treating one symptom (e.g., decoding) while a psychiatrist may target another (e.g., anxiety). The majority of studies of interventions for comorbid RD are with ADHD with few to no studies of other comorbid conditions such as ASD, CD, anxiety, or depression.

### Reading Interventions

Phonics-based reading instruction is the most common and most effective intervention for students with RD (98) and for poor readers (99). Phonics instruction that is systematic and explicit has the greatest evidence (100). Instruction designed to explicitly teach adult students to assign selective attention to grapheme–phoneme associations—as opposed to attempts to memorize whole unfamiliar words—impacts brain circuitry that can subsequently be recruited during reading (101). Reading interventions are effective for students with and without RD when administered by teachers or researchers (102). Although music education has also been investigated as a way to improve reading in children with RD, evidence does not currently support its effectiveness (103).

Reading interventions in comorbid ADHD + RD are shown to be effective regardless of adjunctive ADHD medications (104). In a recent paper, ADHD treatment alone resulted in greater reduction in ADHD symptoms than reading treatment alone, and reading treatment led to greater improvements in reading outcome (word reading and decoding) than ADHD treatment only. The administration of both treatments simultaneously did not result in a greater level of improvement of each outcome (ADHD symptoms and reading skills). In other words, there was no additive value to combining treatments. However, the combined treatment enabled remediation of both ADHD and reading symptoms in the comorbid group simultaneously, so would still be recommended over treating each disorder in isolation (105). It should be noted that this study involved predominantly African American males and should be replicated with a diverse range of demographics.

To be most effective, children with RD and comorbid conditions may need reading interventions to be more specific or combined with other interventions. For example, children with RD + MD who received both reading intervention and number combination intervention outperformed RD + MD students who received reading intervention alone (106). Reading intervention may also need to specifically target the unique reading profiles of subjects with comorbidities. Children with ASD and comorbid reading problems show a profile of intact decoding abilities, yet low reading comprehension, and accordingly, reading intervention specifically targeting vocabulary skills is shown to be most effective in this population (107, 108).

### Socioemotional Health

Because children with RD may be exposed to significant stressors, and RD can co-occur with anxiety and depression, treatments should address socioemotional health in addition to reading. Protective factors that foster resilience for children and adolescents with RD include self-advocacy tools, strength identification, and social connections (109). However, research on evidence-based treatments for depression and anxiety that commonly occur with RD is inadequate and is a critically important area for future work. Cognitive behavioral therapy (CBT), a treatment that focuses on altering negative behavioral and thought patterns, may reduce symptoms of comorbid anxiety and depression in RD children. CBT is the standard for treating unidimensional cases of anxiety and depressive disorders (110, 111) and is shown to be effective in treating psychiatric comorbidities in other conditions that co-occur with RD, such as ADHD (112) and ASD (113). More research is needed to delineate unique modifications that might be necessary for the greatest effectiveness when the emotional condition is combined with RD.

Mindfulness meditation shows increasing promise for benefit to socioemotional health in people with these combined disorders. Mindfulness meditation is shown to reduce anxiety in RD adolescents (114). It is also shown to improve attention and lexical processing/word reading (but not non-word decoding) in combined RD and ADHD in adults, more so than in those with RD only (115). A mindfulness intervention incorporating elements of CBT was shown to improve ODD and CD symptoms in RD + ADHD adolescents, as well as reduce anxiety in RD + anxiety adolescents. Academic performance is thought to be improved through the reduction in anxiety as a result from mindfulness meditation among youth with RD and comorbid conditions (114).

### Biomedical and Nutritional

Pharmacotherapy is increasingly investigated for combined RD and comorbid conditions, although the most common treatment for RD alone is reading interventions. The great majority of these studies examined RD with comorbid ADHD. Results from these studies are summarized in recent reviews (56, 116). In summary, these studies have investigated the use of atomoxetine (ATX), methylphenidate (MPH), and nutritional supplements such as polyunsaturated fatty acids (117) on outcomes of reading, ADHD symptoms, and executive functions in ADHD + RD groups. Reviews reporting on treatment studies found that outcome effect sizes range from small to medium [as low as 0.13 for ATX and as high as 0.60 for MPH (56)], although effects on ADHD symptoms are larger and more consistent than for executive function or reading (56, 116). Future work in this area should investigate the impact of these and other medications on RD with other commonly co-occurring conditions, as well as examine the neurophysiological mechanisms of these treatments in comorbid groups.

### Experimental Interventions

Initial research suggests that neurofeedback training to increase attention processes (118, 119) may be effective in reducing ADHD and RD symptoms, although investigations of these brain-based interventions are too preliminary to be fully endorsed as treatments for RD. Altering cortical excitability using neuromodulation techniques, transcranial magnetic stimulation, and transcranial direct current stimulation is shown to change reading and reading-related abilities in typical and RD adults and children, though parameters such as stimulation frequency and location are not consistent in their benefits (120, 121). These studies have not investigated neuromodulation with RD and comorbid conditions and are still in experimental and proof-of-concept stages.

## Clinical Implications and Significance

Knowledge and awareness of RD are highly relevant to health-care professionals working with children, as mental disorders may be comorbid or blended, and RD can be overlooked or undertreated. Evidence for the co-occurring disorder may be recognized before the RD is identified (e.g., ADHD and ASD), may follow the RD (e.g., depression), or may be intertwined with RD (e.g., anxiety and behavioral disorders). In all of these co-morbidities, the mechanisms of the disorders may overlap, and more research is needed to identify the mechanism of the overlap, the sequencing of their developmental and neurodevelopmental influence, the most beneficial targeting and nature of interventions, and the economic burden of RD with and without treated and untreated comorbid mental disorders. Although one disorder may be identified as the primary target for intervention, comprehensive interventions should address both the RD and the comorbidity to produce optimal treatment results.

## Author Contributions

RH designed the article and wrote the Sections “Introduction” and “Treatment.” RH and SH cowrote the Comorbid Mental Disorders sections—SH also constructed the table. JB wrote the Section “Socioemotional Health.” NW wrote the Section “Reading Interventions.” FH wrote the Section “Overview of RD” and added to all sections. All the authors read and approved the paper.

## Conflict of Interest Statement

Research was conducted in the absence of any commercial or financial relationships that could be construed as a potential conflict of interest.
